# COGNITIVE IMPAIRMENT AMONG OLDER ADULTS WITH INTERSECTING SEXUAL, GENDER, RACIAL, AND ETHNIC MINORITY IDENTITIES

**DOI:** 10.1093/geroni/igad104.0707

**Published:** 2023-12-21

**Authors:** Hyun-Jun Kim, Austin Oswald, Hailey Jung, Karen Fredriksen-Goldsen

**Affiliations:** University of Washington, Seattle, Washington, United States; University of Washington, Seattle, Washington, United States; University of Washington, Seattle, Washington, United States; University of Washington, Seattle, Washington, United States

## Abstract

Emerging research shows Lesbian, gay, bisexual, transgender, and queer (LGBTQ) older adults experience higher prevalence of cognitive impairment when compared to their heterosexual and cisgender peers of a similar age. The same is true for racial and ethnic minority older adults when compared to their White counterparts. Little is known about the cognitive health of older adults with intersecting sexual, gender, racial, and ethnic minority identities, highlighting a critical gap in gerontological research that requires further investigation. This study utilizes four time-point data from the first longitudinal study of a racially and ethnically diverse sample of LGBTQ older adults. Multilevel mixed models accounting for intersecting sexual, gender, racial, and ethnic identities were applied to examine subgroup differences in cognitive impairment as well as risk and protective factors after controlling for confounding factors. Black and Hispanic LGBTQ minority older adults (b=5.20, p<.01; b=4.05, p<.01) experience higher levels of cognitive impairment over time when compared to their White LGBTQ peers. Day-to-day discrimination targeting the confluence of sexual/gender and racial/ethnic minority status (b=2.78, p<.05) was positively associated with higher cognitive impairment. Community engagement (b=-.46, p<.05) was negatively associated with cognitive impairment regardless of race and ethnicity whereas network size was exclusively negatively associated with cognitive impairment for Black LGBTQ older adults. The overall findings demonstrate racial and ethnic differences in cognitive impairment among LGBTQ older adults and illuminate the importance of culturally tailored interventions that account for intersectionality, social connectivity, and discrimination to promote cognitive health for specific subgroups.

